# Raw and processed data used in non-covalent functionalization of single walled carbon nanotubes with Co-porphyrin and Co-phthalocyanine and its effect on field-effect transistor characteristics

**DOI:** 10.1016/j.dib.2021.107366

**Published:** 2021-09-14

**Authors:** Fatima Bouanis, Mohamed Bensifia, Ileana Florea, Samia Mahouche-Chergui, Benjamin Carbonnier, Daniel Grande, Céline Léonard, Abderrahim Yassar, Didier Pribat

**Affiliations:** a COSYS-LISIS, Université Gustave Eiffel, IFSTTAR, Marne-la-Vallée F-77454, France; b Laboratory of Physics of Interfaces and Thin Films, UMR 7647 CNRS/ Ecole Polytechnique, IPParis, Palaiseau 91128, France; c MSME, Université Gustave Eiffel, CNRS UMR 8208, Université Paris-Est Créteil, Marne-la-Vallée F-77454, France; d CNRS, ICMPE, UMR 7182, Université Paris-Est Créteil, 2 rue Henri Dunant, Thiais 94320, France

**Keywords:** SWNTs, CNTFETs, Noncovalent functionalization, Porphyrin, Phthalocyanine

## Abstract

This scientific data article is related to the research work entitled “Non-Covalent functionalization of Single Walled Carbon Nanotubes with Fe-/Co-porphyrin and Co-phthalocyanine for Field-Effect Transistor Applications” published in “Organic electronics” (10.1016/j.orgel.2021.106212). In this work, we present the data of morphological, chemical and structural analyses of non-covalent functionalization of SWNTs with Co-porphyrin and Co-phthalocyanine. The analyses were performed by Raman spectroscopy, transmission electron microscopy as well as the electrical characterization of CNTFETs. This work is completed by the data of the theoretical calculations performed using Density Functional Theory (DFT).

**Specifications Table**

**Table:** 

Subject	Materials science and engineering
Specific subject area	Non-covalent functionalization, materials and electrical properties
Type of data	Table Image Graph Figure
How data were acquired	Data were acquired using spectroscopy Raman, TEM, electrical measurements, and theoretical calculations were performed by DFT. Instruments: OriginPro 8.5 software, CRYSTAL17 code
Data format	Data Raw and processed Analyzed Filtered
Parameters for data collection	All data were collected before and after the functionalization of SWNTs with Co-Por (10^−4^ mol/l in DCM) and Co-Phc (5.10^−3^ mol/l in DMF) at room temperature.
Description of data collection	A solution of molecules was deposited on the surface of SWNTs, followed by evaporation/drying at 40 °C for 1 h. After washing several times with DCM or DMF, the SWNTs were analysed using Raman spectroscopy, TEM and electrical measurements. The analyses were completed by DFT calculations. The data were compared with pristine SWNTs to confirm the non-covalent functionalization.
Data source location	Institution: Laboratory of Physics of Interfaces and Thin Films, UMR 7647 CNRS/ Ecole Polytechnique, IPParis City/Town/Region: 91,128 Palaiseau Country: France Latitude and longitude (and GPS coordinates, if possible) for collected samples/data:
Data accessibility	Data are available with the article.
Related research article	Fatima Z. Bouanis, Mohamed Bensifia, Ileana Florea, Samia Mahouche-Chergui, Benjamin Carbonnier, Daniel Grande, Céline Léonard, Abderrahim Yassar, Didier Pribat, published in Organic Electronics (https://doi.org/10.1016/j.orgel.2021.106212).

**Value of the Data**

• Morphological and electrical investigations of novel nano-hybrid materials based on SWNTs functionalized with appropriate molecules led to development of new devices with potential applications in sensing.
• Morphological characterization of functionalized SWNTs at nanoscale, chemical mapping with nanoscale resolution and understanding the interfacial interactions between adsorbed molecules and SWNTs to facilitates their implementation as a sensing layer.
• The data can used to obtain an accurate chemical, structural and morphological information of nano-hybrid materials and then to understanding the relationship between molecular structure, functionalization and electrical properties.
• These data are useful to a better understanding the work related to the publication as well as to whom interested to reproduce or to verify the findings.
• The present data are of importance in characterizing non-covalent functionalization of carbon nanotube.
• These data provide a scientific assessment of non-covalent functionalization SWNTs with п-conjugated molecules.

## 1 Data Description

SWNTs were synthesized from Ru catalysts on silicon substrates using d-HFCVD, then functionalized with 5,10,15,20-Tetraphenyl-21H,23H-porphine cobalt(II) (Co-Por) and cobalt (II) phthalocyanin (Co-Phc). A solution of molecules (10^−4^ mol/l Co-Por or 5.10^−3^ mol/l of Co-Phc in DCM or DMF) was deposited directly on the surface of SWNTs followed by evaporation/drying at 40 °C for 1 h. After rinsing several times with DCM or DMF, the new nano-hybrid materials were first characterized with Raman spectroscopy.

**Fig. 1:**
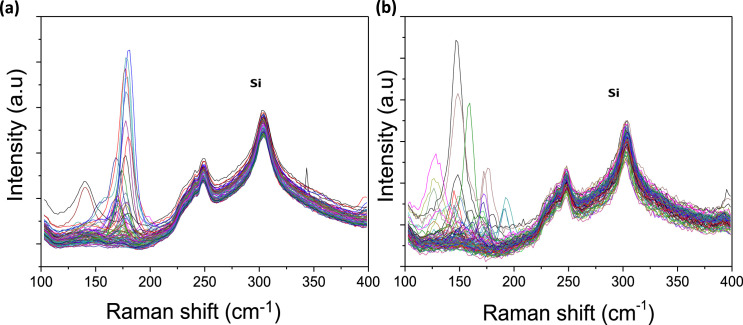
(a) Raman radial breathing mode (RBM) spectra of SWNTs synthesized from a 0.05 nm-thick Ru layer at 850 °C under atomic hydrogen flux (H_at_). (b) RBM spectra of SWNTs functionalized with Co-Po.

**Fig. 2:**
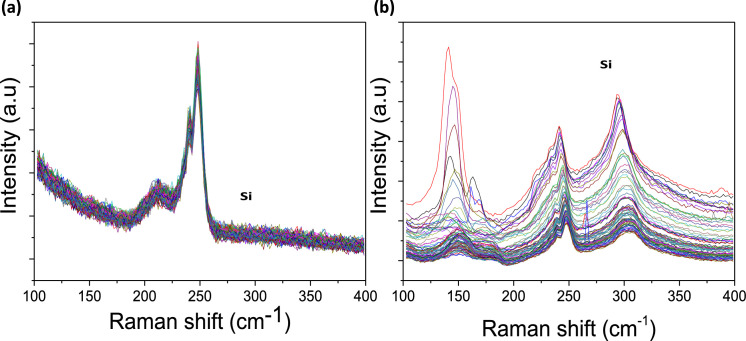
RBM signals of as grown SWNTs synthesized from Ru nanoparticles (a) and after functionalization with Co-Phc (b).

**Fig. 3:**
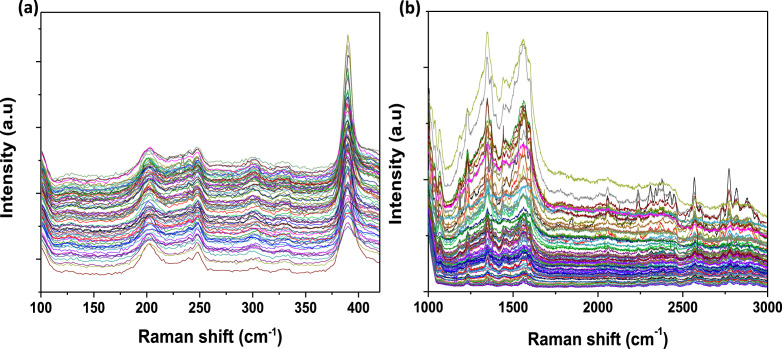
Raman mapping of an oxidized silicon substrate (SiO_2_/Si) functionalized with Co-Por in the low-frequency region (a) and high-frequency region (b).

**Fig. 4:**
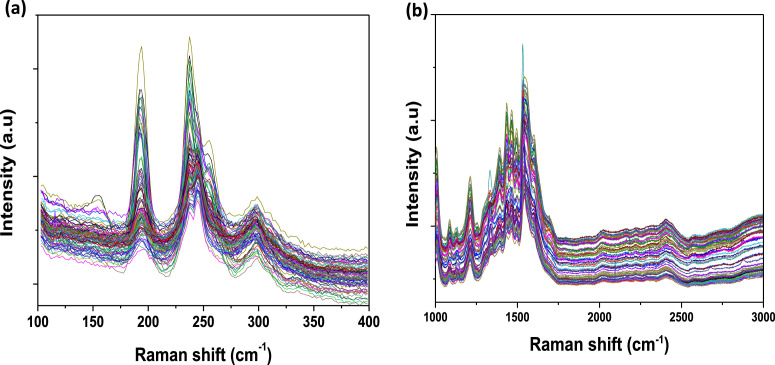
(a) Raman mapping of SiO_2_/Si functionalized with Co-Phc in the low-frequency region and (b) high-frequency region.

**Fig. 5:**
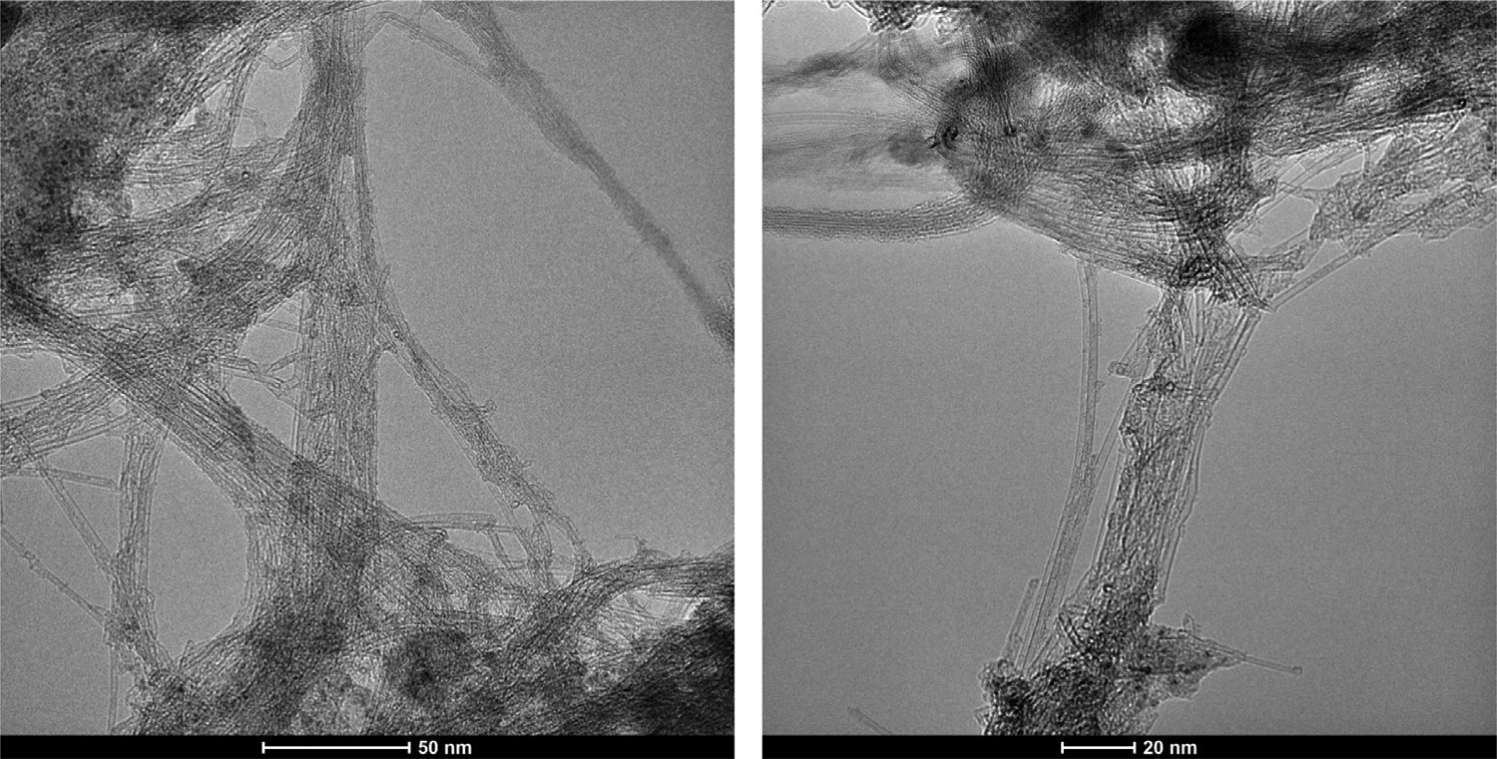
TEM images of SWNTs synthesized from 0.05 nm Ru layer at 850 °C under atomic hydrogen (H_at_).

**Fig. 6:**
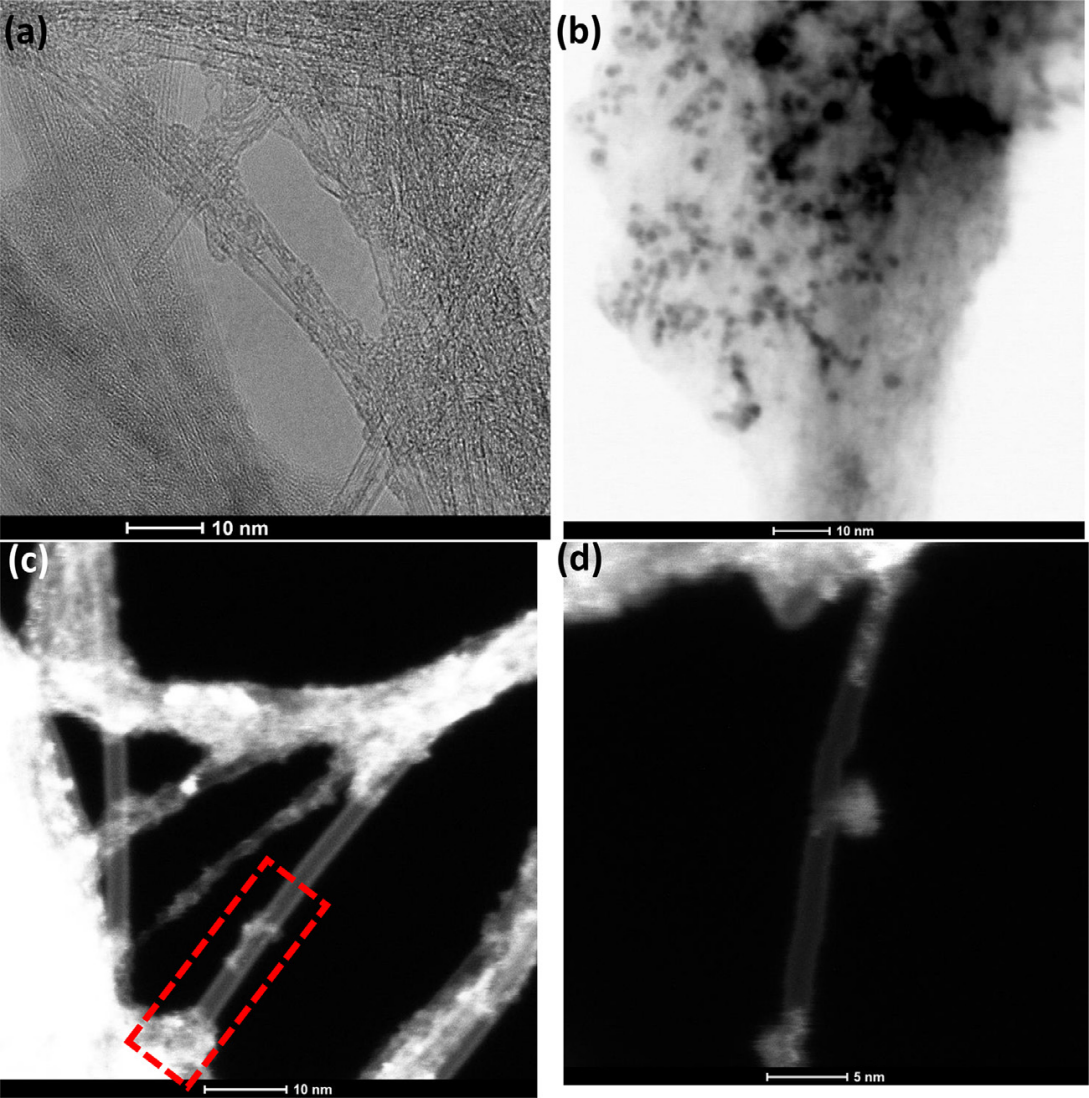
TEM images of SWNTs functionalized with Co-Por. (a) as grown' SWNTs. (b) HR-STEM-BF of Co-Por complex. (c) STEM-HAADF image of functionalized SWNTs with Co-Por. (d) HR-STEM-HAADF image zoom of the area marked by the red dotted rectangle in (c) showing functionalized SWNTs with Co-Por.

**Fig. 7:**
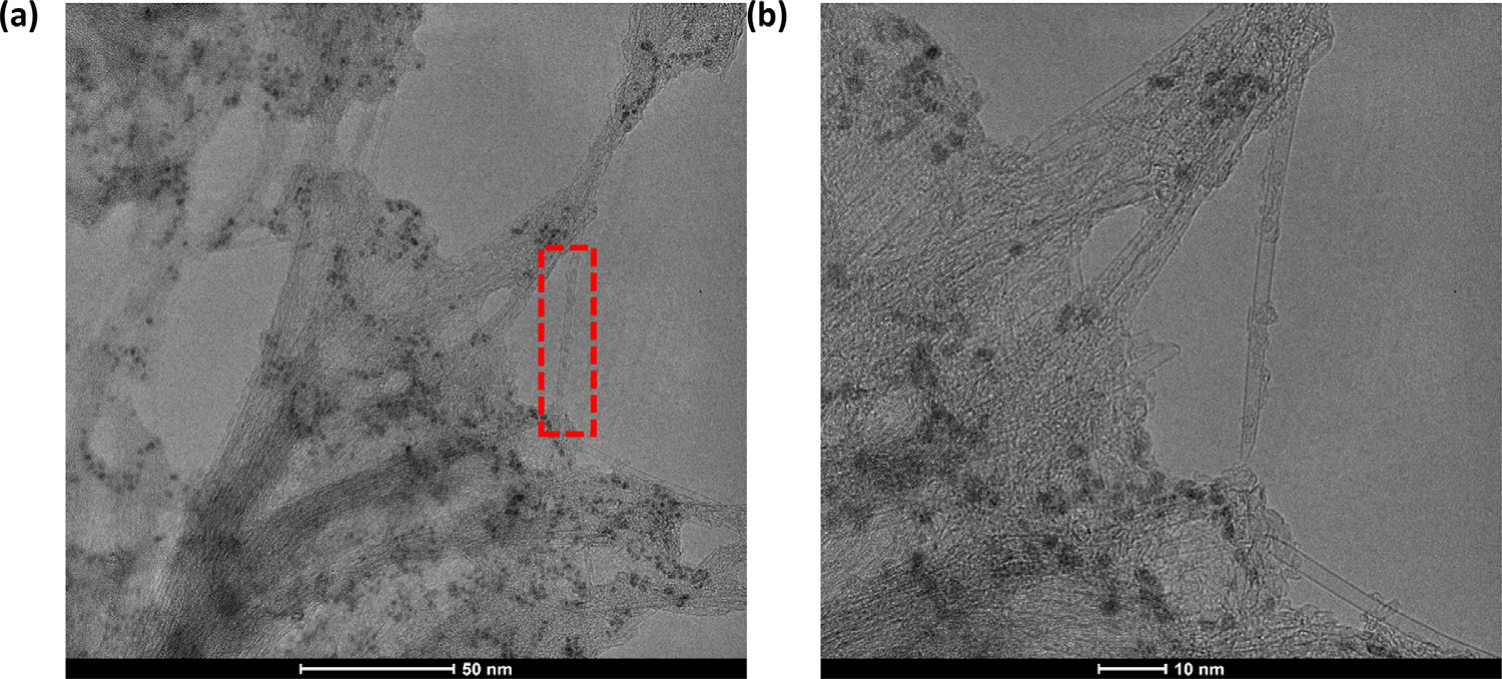
TEM images of SWNTs functionalized with Co-Phc. (a) HR-TEM image of functionalized SWNTs with Co- Phc. (b) HR-TEM image zoom of the area marked by the red dotted rectangle in (a) showing one functionalized SWNT with Co-Phc.

**Fig. 8:**
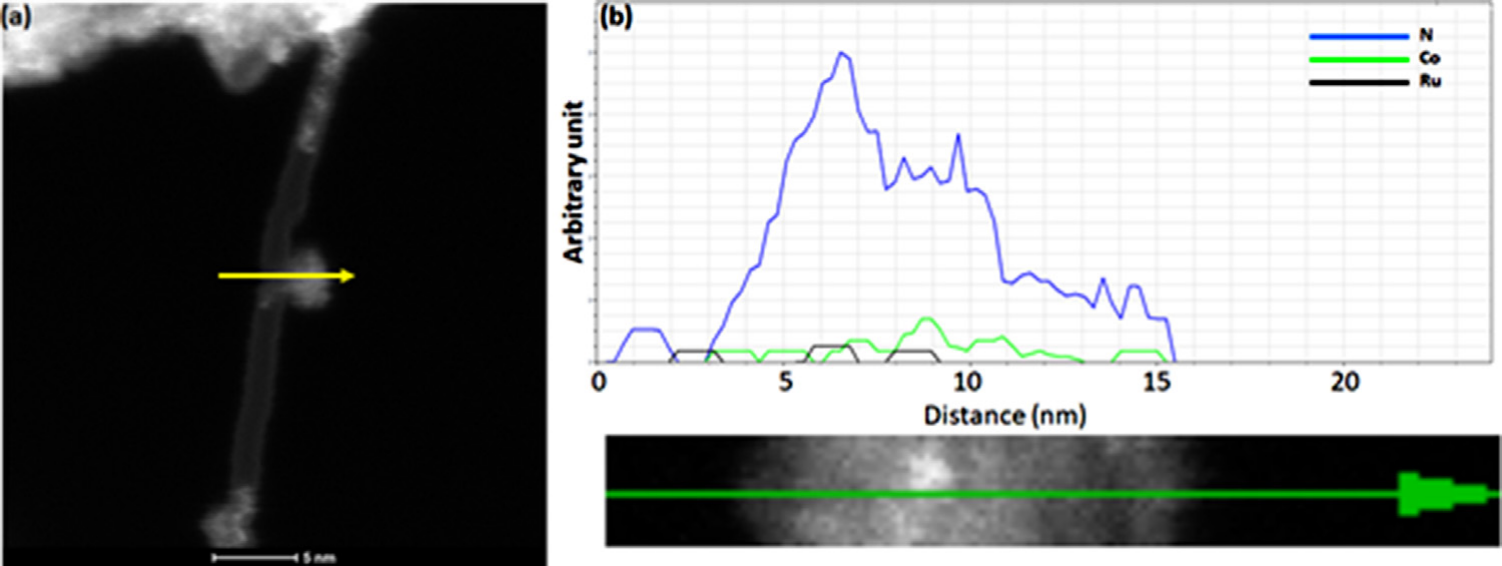
STEM-HAADF-EDS line scan analysis on individual functionalized SWNTs with Co-Por: **(a**) STEM-HAADF micrograph of the chosen area for the analysis. (**b**) EDS-STEM line scan with the Nitrogen (in blue), Cobalt (in green) and Ruthenium (in black) concentrations recorded along the yellow arrow in (a). (For interpretation of the references to color in this figure legend, the reader is referred to the web version of this article.)

**Fig. 9:**
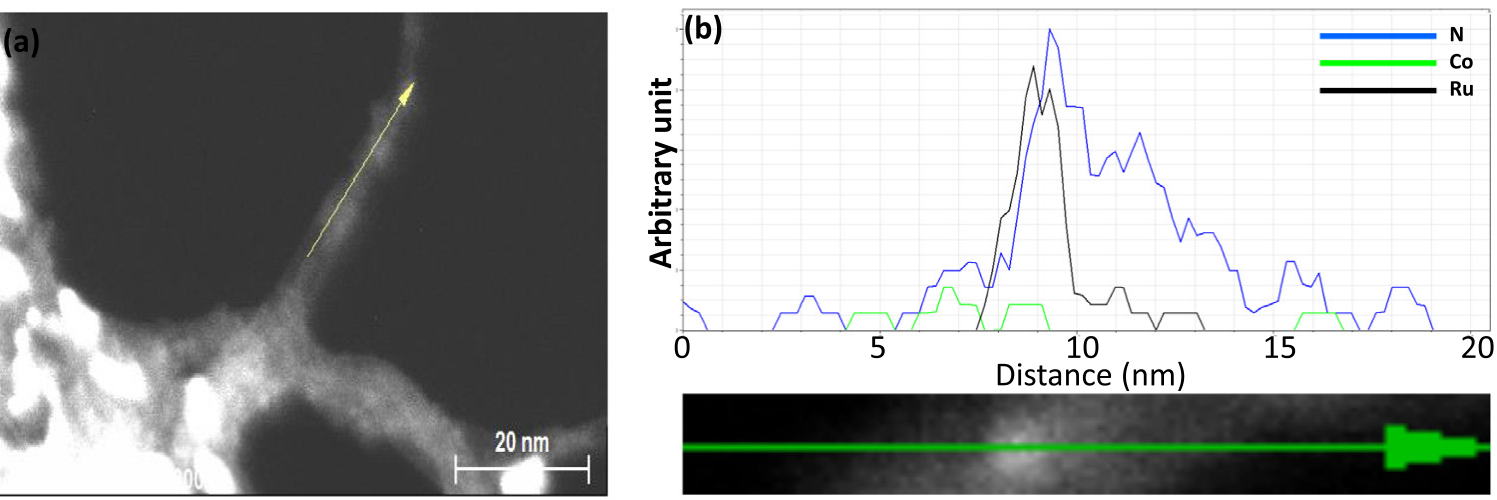
EDS-STEM chemical analysis on individual functionalized SWNTs with Co-Phc: **(a**) STEM-HAADF micrograph on the chosen area for the analysis. (**b**) EDS-STEM line scan with the Nitrogen (in blue), Cobalt (in green) and Ruthenium (in black) concentrations recorded along the yellow arrow in (a). (For interpretation of the references to color in this figure legend, the reader is referred to the web version of this article.)

**Fig. 10:**
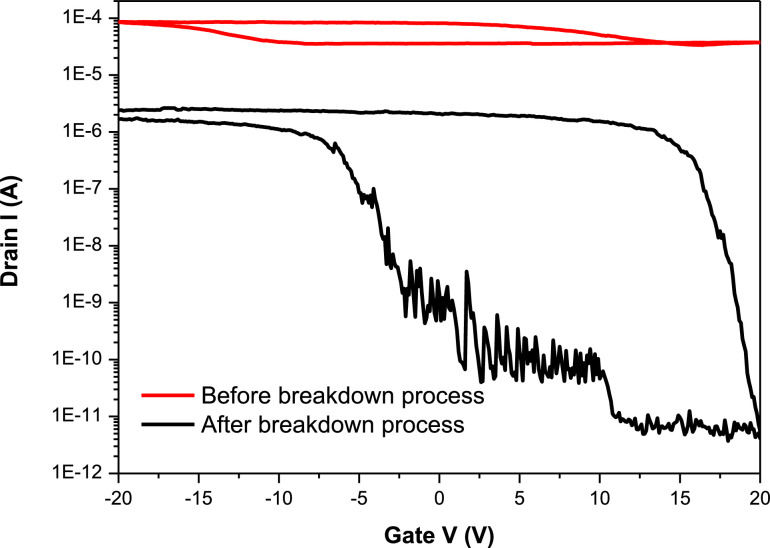
Plots of drain current (*I*_ds_) versus gate voltage (*V*_g_) of CNTFET device before (red curve) and after (black curve) breakdown process. (For interpretation of the references to color in this figure legend, the reader is referred to the web version of this article.)

**Fig. 11:**
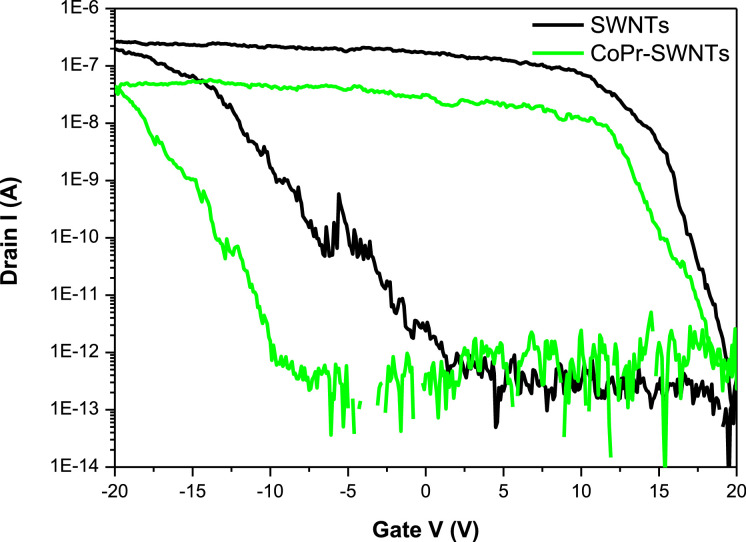
*I*_ds_ versus *V*_g_ characteristics of CNTFET before (black curve) and after (green curve) functionalization with Co-Por. *V*_ds_ = 2 V. (For interpretation of the references to color in this figure legend, the reader is referred to the web version of this article).

**Fig. 12:**
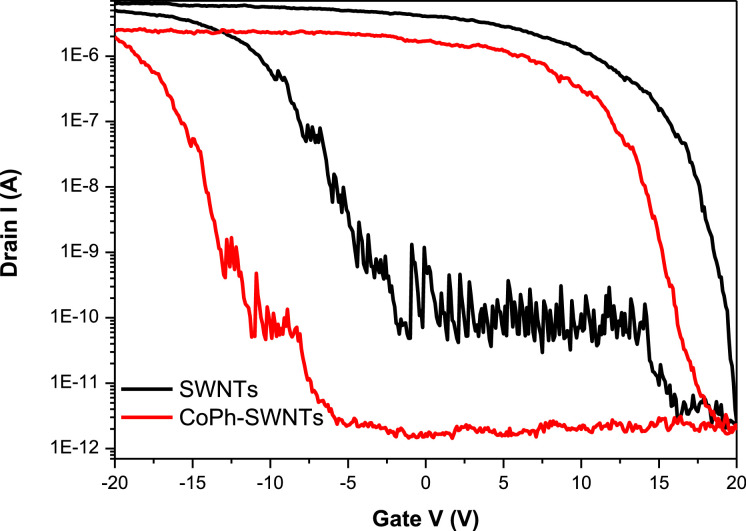
*I*_ds_ vs *V*_g_ characteristics of CNTFET before (black curve) and after (red curve) modification with Co-Phc. *V*_ds_ = 2 V. (For interpretation of the references to color in this figure legend, the reader is referred to the web version of this article.)

Fig. 1 presents Raman radial breathing mode (RBM) of SWNTs synthesized from a 0.05 nm-thick Ru layer at 850 °C under atomic hydrogen flux (H_at_). (b) RBM spectra of SWNTs functionalized with Co-Po. and Fig. 2 shows RBM signals of as grown SWNTs synthesized from Ru nanoparticles (a) and after functionalization with Co-Phc (b). For comparison, the Raman spectra of functionalized silicon substrates (SiO_2_/Si) were presented in Figs. 3 and 4. Fig. 3 shows the Raman mapping of an oxidized silicon substrate (SiO_2_/Si) functionalized with Co-Por in the low-frequency region (a) and high-frequency region (b). Fig. 4 is a Raman mapping of SiO_2_/Si functionalized with Co-Phc in the low-frequency region (a) and high-frequency region (b). The morphological analysis were performed by TEM. Fig. 5 shows TEM images of SWNTs synthesized from 0.05 nm Ru layer at 850 °C under atomic hydrogen (H_at_). Fig. 6 presents TEM images of SWNTs functionalized with Co-Por: (a) as grown’ SWNTs, (b) HR-STEM-BF of Co-Por complex, (c) STEM-HAADF image of functionalized SWNTs with Co-Por, (d) HR-STEM-HAADF image zoom of the area marked by the red dotted rectangle in (c) showing functionalized SWNTs with Co-Por. Fig. 7 shows TEM images of SWNTs functionalized with Co-Phc: (a) HR-TEM image of functionalized SWNTs with Co-Phc, (b) HR-TEM image zoom of the area marked by the red dotted rectangle in (a) showing one functionalized SWNT with Co-Phc. Fig. 8 shows STEM-HAADF-EDS line scan analysis on individual functionalized SWNTs with Co-Por: (a) STEM-HAADF micrograph of the chosen area for the analysis, (b) EDS-STEM line scan with the Nitrogen (in blue), Cobalt (in green) and Ruthenium (in black) concentrations recorded along the yellow arrow in (a). Fig. 9 represents EDS-STEM chemical analysis on individual functionalized SWNTs with Co-Phc: (a) STEM-HAADF micrograph on the chosen area for the analysis, (b) EDS-STEM line scan with the Nitrogen (in blue), Cobalt (in green) and Ruthenium (in black) concentrations recorded along the yellow arrow in (a). The theoretical calculations were performed using DFT. In Table 1, the binding energies (Eb in kJ/mol) and minimal distances between Co and a C atom of the SWNT (d_m_ in Å) at the PBE0-D3/6–31G** level of theory using CRYSTAL are given (using Eq. (1) in SI of the related research article). Table 2 the net electron transfer (∆q) from Mulliken analysis at PBE0-D3/6–31G** level of theory using CRYSTAL [5]. The data were confirmed by electrical measurements. Fig. 10 presents plots of drain current (*I_ds_*) versus gate voltage (*V_g_*) of CNTFET device before (red curve) and after (black curve) breakdown process. Fig. 11 shows *I_ds_* versus *V_g_* characteristics of CNTFET before (black curve) and after (green curve) functionalization with Co-Por. *V_ds_* = 2 V. Fig. 12 represents *I_ds_* vs *V*_g_ characteristics of CNTFET before (black curve) and after (red curve) modification with Co-Phc. *V*_ds_ = 2 V.

**Table 1:** The binding energies (E_b_ in kJ/mol) and minimal distances between Co and a C atom of the SWNT (d_m_ in Å) at the PBE0-D3/6–31G** level of theory using CRYSTAL [5].

System	E_b_ (kJ/mol)	d_m_ (Å)
SWNT (8,0)_Co-Phc	−124.69	3.02
SWNT (8,0)_Co-Por	−125.74	3.27

**Table 2:** The net electron transfer (∆q) from Mulliken analysis at the PBE0-D3/6–31G**level of theory using CRYSTAL [5].

System A_B	Δq(A) (e)	Δq(B) (e)	Δq(A)+ Δq(B)
SWNT (8,0)_Co-Phc	0.0571221	0.0571226	5E-07
SWNT (8,0)_Co-Por	−0.0495356	0.0495362	6E-07

## 2 Experimental Design, Materials and Methods

### 2.1 Materials

The SWNTs were synthesized on SiO_2_/Si substrates from thin evaporated Ru films (0.05 nm of Ru film thickness) [1]. The Ru catalyst was deposited by ultra-high vacuum evaporation, similar to Molecular Beam Epitaxy (MBE). The SWNTs synthesis was performed using a homemade double hot filament CVD (d-HFCVD) system.

5,10,15,20-Tetraphenyl-21H,23H-porphine cobalt(II) (Co-Por, 85%) and cobalt (II) phthalocyanin (Co-Phc, 97%) were purchased from ``Merck''. The reagents were used without further purification.

### 2.2 Methods

1. The functionalization of SWNTs was achieved following our previously published procedures [2].
2. The Raman mapping was performed, using a laser excitation of λ=532 nm with 10 s acquisition time and 5 accumulations per spectrum. Raman mapping was performed on 15 µm × 15 µm areas with a step size of 0.5 µm.
3. The HR-TEM analyses were performed using a FEI-Titan electron microscope operating at 80 and 300 kV. TEM grids were prepared following the process described in [3].
4. Energy dispersive spectroscopy (EDS) elemental analyses at nanoscale resolution of functionalized SWNTs were performed on a 200 kV Titan-Themis TEM/STEM electron microscope equipped with a Cs probe corrector and a Chemistem Super-X detector.
5. Bottom-gate TFTs were fabricated by deposited 40 nm thick source-drain palladium contacts directly on SWNTs, using standard UV lithography, e-beam evaporation and lift-off processes, a detailed account of the fabrication procedure of CNTFET, is described elsewhere [1,4].
6. The electrical characteristics of the CNTFETs were measured using a semiconductor parametric analyzer Keithley 4200-SCS under ambient conditions by applying a source-drain voltage (*V_ds_* = 2 V) and measuring the source-drain current (*I_ds_*) as a function of the source-gate voltage (*V*_g_).
7. Theoretical density functional theory calculations (DFT) (open shell) were carried out using CRYSTAL17 code [5].

## Ethics Statement

Ethics Statement This article does not contain any studies involving animals or human participants performed by any of the authors.

## CRediT Author Statement

**Abderrahim Yassar** and **Didier Pribat:** Writing, Investigation, Validation; **Fatima Bouanis** and **I. Florea:** Data curation, Writing, Software, Investigation, Validation; **Céline Léonard** and **Mohamed Bensifia:** Visualization, Investigation, Software, Writing, Validation; **Samia Mahouche-Chargui, Benjamin Carbonnier** and **Daniel Grande:** Visualization, Validation.

## Declaration of Competing Interest

The authors declare that they have no known competing financial interests or personal relationships which have or could be perceived to have influenced the work reported in this article.

## Appendix Supplementary materials

Image, application 1

Image, application 2

Image, application 3

Image, application 4

Image, application 5

Image, application 6

Image, application 7

Image, application 8

Image, application 9

Image, application 10

Image, application 11

Image, application 12

Image, application 13

Image, application 14
